# Robotic total gastrectomy with thrombectomy and portal vein reconstruction for gastric cancer and portal vein tumor thrombus

**DOI:** 10.1186/s12957-022-02502-8

**Published:** 2022-02-16

**Authors:** Masaaki Yamamoto, Takeshi Omori, Naoki Shinno, Hisashi Hara, Yosuke Mukai, Takahito Sugase, Tomohira Takeoka, Kei Asukai, Takashi Kanemura, Nozomu Nakai, Shinichiro Hasegawa, Keijiro Sugimura, Hirofumi Akita, Naotsugu Haraguchi, Junichi Nishimura, Hiroshi Wada, Hidenori Takahashi, Chu Matsuda, Masayoshi Yasui, Hiroshi Miyata, Masayuki Ohue

**Affiliations:** Department of Gastroenterological Surgery, Osaka International Cancer Institute, 3-1-69 Otemae, Chuo-ku, Osaka, 541-8567 Japan

**Keywords:** Gastric cancer, Portal vein tumor thrombus, Robot, Gastrectomy, Thrombectomy, Portal vein reconstruction

## Abstract

**Background:**

Gastric cancer with portal vein tumor thrombus (PVTT) is poor prognosis, and the treatment remains challenging. Regarding surgery, there are only reports of highly invasive laparotomy. We report some techniques of the completely robotic total gastrectomy with thrombectomy and portal vein reconstruction for the patient with gastric cancer and PVTT for the first time.

**Case presentation:**

A 79-year-old man was diagnosed with a 5-cm gastric cancer on the side of the lesser curvature from the middle of the gastric body to the cardia. Computed tomography revealed a massive PVTT extending from the left gastric vein to the portal trunk (28 x 16 mm). There were no other distant metastases. After 3 cycles of the chemotherapy, the PVTT shrank to 19 x 12 mm. After obtaining informed consent from the patient, robotic total gastrectomy with regional lymphadenectomy and thrombectomy were performed. We used the da Vinci Xi Surgical System. A 3-cm incision was made at the umbilicus, and a wound retractor was placed. Five additional ports were placed. The right side suprapancreatic lymph nodes were performed at the time of the thrombectomy. It was important to identify the precise extent of the PVTT with intraoperative ultrasonography before the thrombectomy. After PVTT identification, the portal trunk was clamped above and below the tumor thrombus with vascular clips. The membrane on the anterior wall of the portal trunk around the PVTT was carefully incised with da Vinci Scissors. The tumor thrombus was completely enucleated without separation. The incised part of the portal trunk was reconstructed with continuous 5-0 synthetic monofilament nonabsorbable polypropylene sutures. After removing the vascular clamps, we made sure there was no leakage from the portal vein and no tumor thrombus remnants with intraoperative ultrasonography. Robotic total gastrectomy with lymphadenectomy and Roux-en-Y reconstruction were performed. The patient was discharged without complications. The patient has remained alive for 30 months after surgery.

**Conclusions:**

Robotic total gastrectomy with thrombectomy and portal vein reconstruction is a safe, minimally invasive, and precise surgery. It may contribute to improved prognosis of gastric cancer with PVTT when combined with chemotherapy.

## Background

Gastric cancer with portal vein tumor thrombus (PVTT) have been reported to have poor prognosis [1–3]. The median survival of gastric cancer patients with PVTT is 5.4 months, and the 5-year survival rate is less than 10% [3]. Several studies have shown that the prognosis of patients with gastric cancer and PVTT are improved after gastrectomy [2–4]. However, there are no reports about patients with gastric cancer and PVTT who undergo laparoscopic or robotic gastrectomy. We reported the first case of a patient with gastric cancer and PVTT who underwent robotic radical total gastrectomy with thrombectomy and portal vein reconstruction after chemotherapy.

## Case presentation

**Fig. 1:**
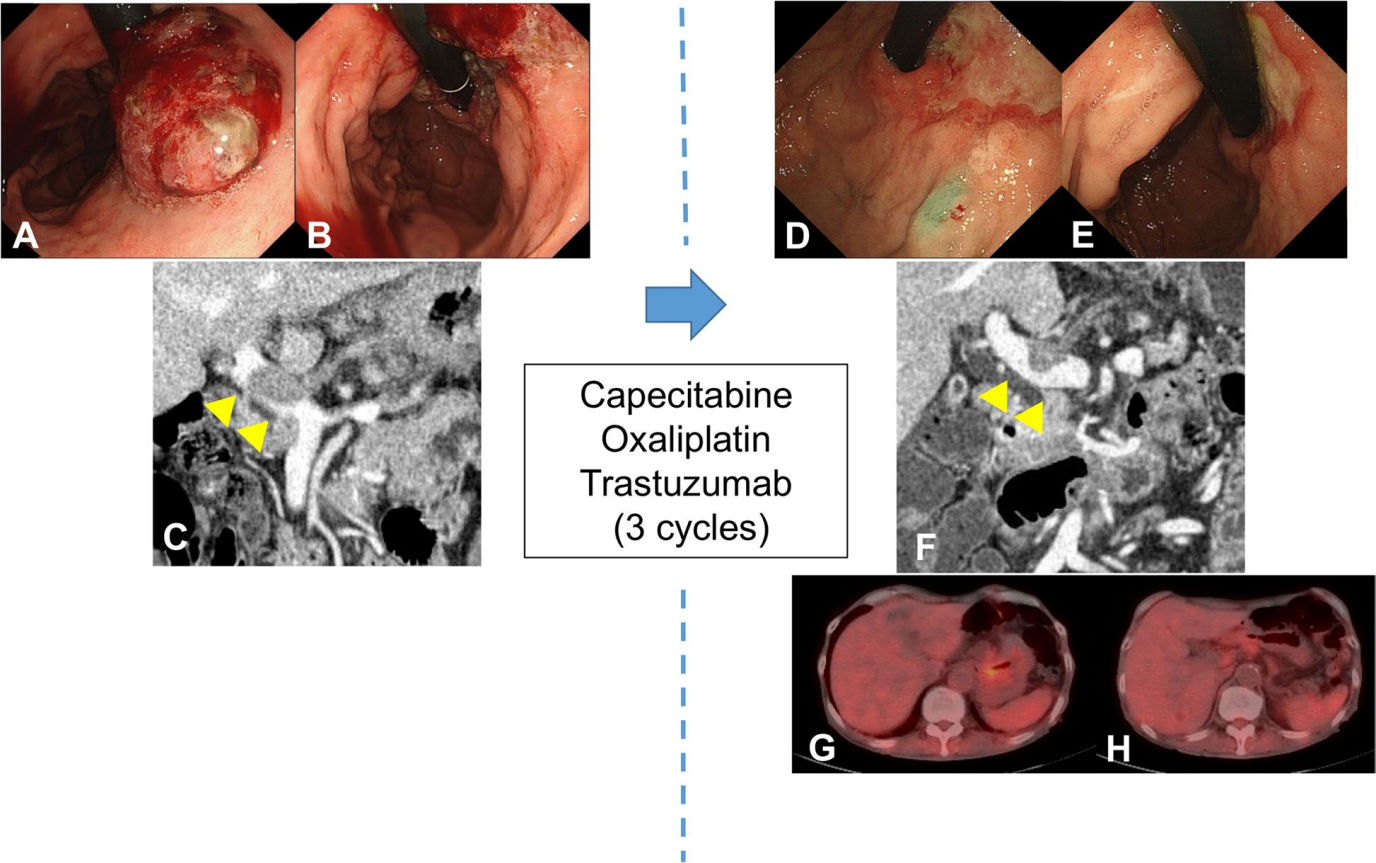
Gastrointestinal endoscopy revealed an elevated tumor on the side of the lesser curvature, from the middle of the gastric body to the cardia. The length of the tumor was approximately 5 cm before chemotherapy (**A**, **B**). Computed tomography revealed portal vein tumor thrombus (PVTT) (**C**). After chemotherapy, the primary gastric lesion flattened and the PVTT shrank (**D–F**). Arrowheads show PVTT (**C**, **F**). Positron emission tomography only showed abnormal uptake in the primary gastric lesion and PVTT (**G**, **H**)

A 79-year-old man presented with upper abdominal pain. He was diagnosed with gastric cancer and referred to our hospital. Gastrointestinal endoscopy revealed an elevated tumor on the side of the lesser curvature from the middle of the gastric body to the cardia. The length was approximately 5 cm (Fig. 1A, B). Pathological examination of a biopsy specimen revealed moderately and poorly differentiated adenocarcinoma and human epidermal growth factor receptor-2 (HER-2) positivity. Enhanced computed tomography (CT) revealed a massive PVTT extending from the left gastric vein to the portal trunk (size: 28.7 x 16.9 mm) (Fig. 1C). There were no other distant metastases. Serum carcinoembryonic antigen and alpha-fetoprotein levels were high, 108 ng/mL and 36 ng/mL, respectively. Carbohydrate antigen 19-9 levels were normal. The patient had no liver diseases.

The treatment regimen mainly consisted of chemotherapy: capecitabine (3000 mg/body/day, oral, days 1–14, followed by 7 days of rest), oxaliplatin (130 mg/m^2^, intravenous, day 1), and trastuzumab (initial dose 8 mg/kg, subsequent doses 6 mg/kg, intravenous, day 1). After 3 cycles of the regimen, the primary gastric lesion was flattened and the PVTT shrank to 19.7 x 12.0 mm (Fig. 1D–F). Positron emission tomography (PET) revealed abnormal uptake only in the primary gastric lesion and PVTT (Fig. 1G, H). The therapeutic effect was evaluated as partial response (PR) according to the Response Evaluation Criteria in Solid Tumors (RECIST), version 1. 1[5]. Since the patient experienced grade 3 anorexia and diarrhea based on the Common Terminology Criteria for Adverse Events (CTCAE), version 5.0 during the chemotherapy, it was hard for him to continue with this chemotherapy regimen. Therefore, after obtaining informed consent from the patient, robotic total gastrectomy with regional lymphadenectomy and thrombectomy was performed after the three courses of chemotherapy.

### Operative technique

**Fig. 2:**
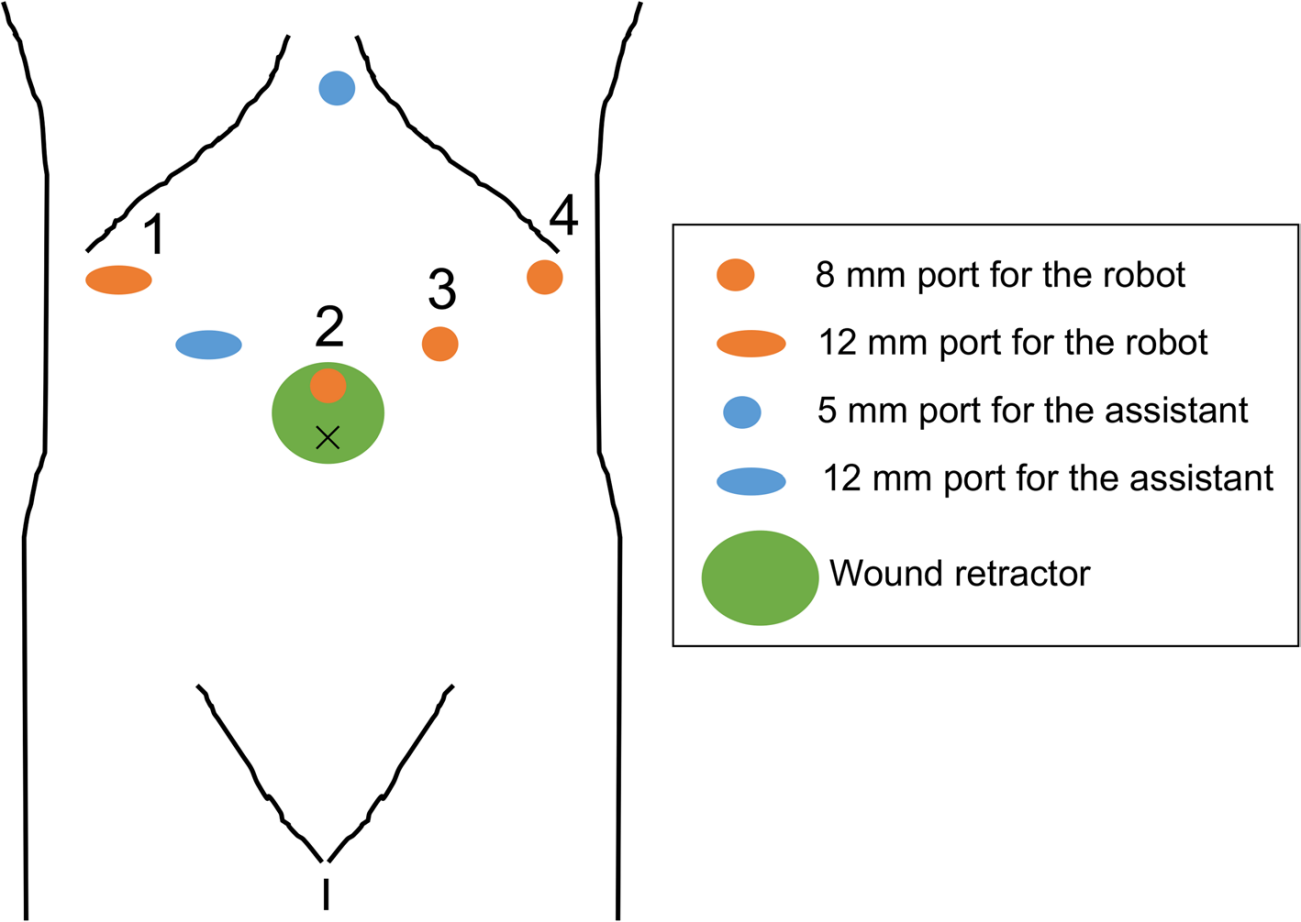
Port placement for robotic total gastrectomy with thrombectomy and portal vein reconstruction. The numbers refer to robot arms

We used the da Vinci Xi Surgical System (Intuitive Surgical, Inc., CA, USA). Under general anesthesia, the patient was positioned in a head-up tilt position (15°). A 3-cm incision was made at the umbilicus and a wound retractor was placed. The first port (8 mm) for the camera was inserted through the wound retractor (Hakko Co., Ltd., Tokyo, Japan). Five additional ports were inserted as follows: 12 mm port for the robot in the right hypochondrium, 12 mm port for the assistant surgeon in the right upper abdomen between the 12 mm port for robot and the umbilicus, 8 mm port for the robot in the left hypochondrium, 8 mm port for the robot in the left upper abdomen between the 8 mm port for the robot in the hypochondrium and the umbilicus, and 5 mm port for the assistant surgeon (AirSeal®, ConMed, Largo, FL, USA) in the epigastric region. The first, second, third, and fourth robotic arms were docked on the 12-mm right hypochondrium, camera, left upper, and left hypochondrium ports, respectively (Fig. 2). Peritoneal and liver metastases were not detected. Intraoperative cytodiagnosis of ascites fluid was negative. Lymph node dissection was performed according to the criteria in the gastric cancer guidelines of the Japanese Gastric Cancer Association [6, 7].

We firstly start to perform lymphadenectomy at the lymph nodes along the distal portion of the stomach (no. 4sb, 4d, 5, 6) followed by resect duodenum. Next, the lymphadenectomy is performed at the left side suprapancreatic nodes (no. 7, 9, 11p, 11d). The dissection of the right side suprapancreatic nodes (no. 8a, 12a) are performed at the time of the thrombectomy.

**Fig. 3:**
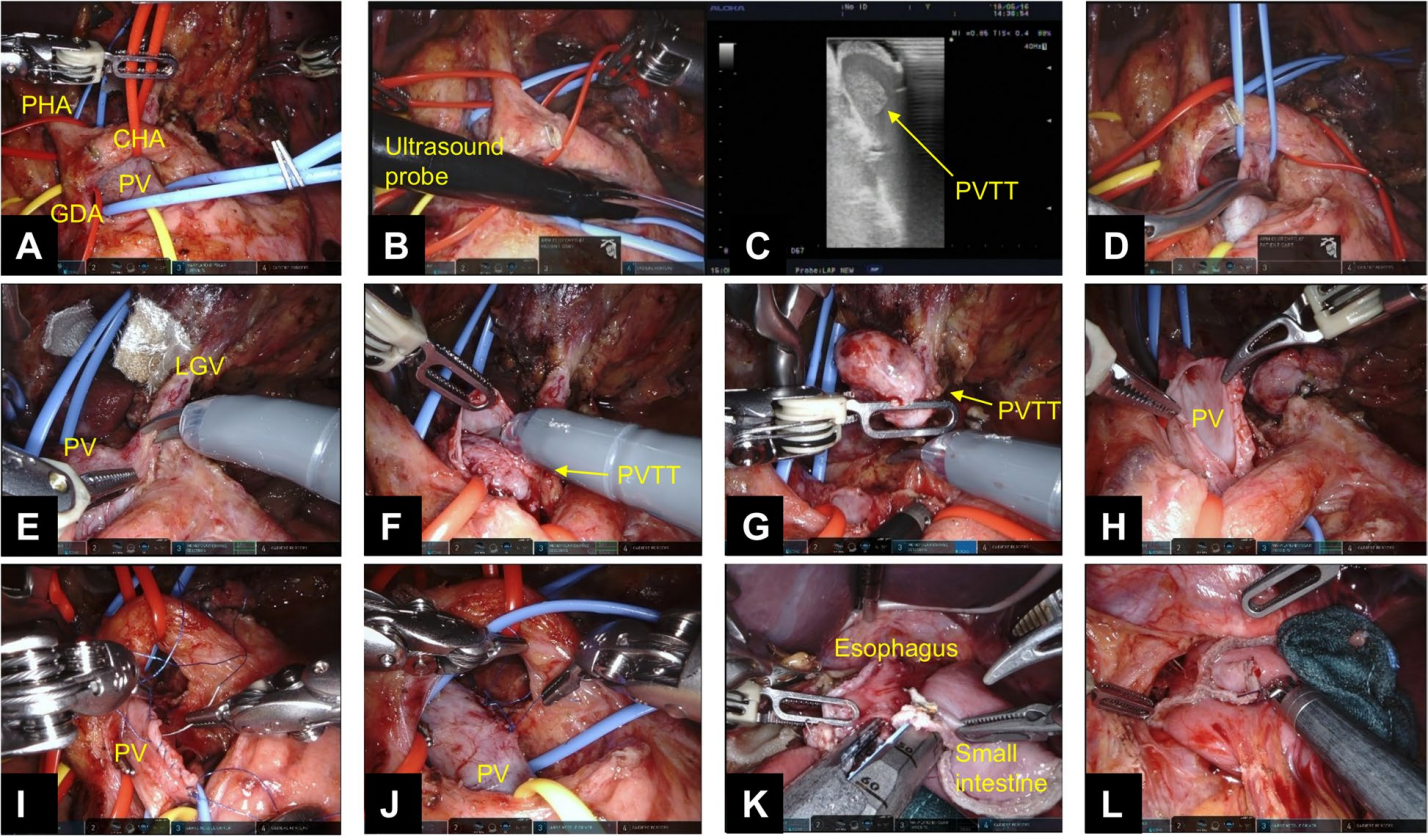
**A**–**I** Surgical procedure for thrombectomy and portal vein reconstruction (**A–J**). A side-to-side esophagojejunal anastomosis was created with the linear stapler. The entry hole of the esophagojejunal anastomosis was closed with the linear stapler (**K**, **L**). Abbreviations: PHA proper hepatic artery, CHA common hepatic artery, PV portal vein, GDA gastroduodenal artery, PVTT portal vein tumor thrombus, LGV left gastric vein

Regarding thrombectomy, the common hepatic artery, proper hepatic artery, gastroduodenal artery, PV, superior mesenteric vein, and left gastric vein (LGV) were definitively identified and labeled with vascular tape (Fig. 3A). The precise extent of the PVTT, from the LGV to the portal trunk, was identified with intraoperative ultrasonography (Fig. 3B, C). After PVTT identification, the portal trunk was clamped above and below the tumor thrombus with vascular clips (Fig. 3D). The membrane on the anterior wall of the portal trunk around the LGV bifurcation was carefully incised with da Vinci Scissors. The incision was gradually extended along the tumor thrombus (Fig. 3E, F). The tumor thrombus was completely enucleated without separation (Fig. 3G, H). The incised part of the portal trunk was reconstructed with continuous 5-0 synthetic monofilament nonabsorbable polypropylene sutures (Fig. 3I). After removing the vascular clamps, we made sure there was no leakage from the PV and no tumor thrombus remnants with intraoperative ultrasonography (Fig. 3J).

After the thrombectomy and PV reconstruction, the lymphadenectomy of the paracardial nodes (no. 1, 2, 3, 4sa) are performed and resect the esophagus. Total gastrectomy and D2 lymphadenectomy are the same as previously described [8, 9]. After the resection of the stomach, it was removed from the abdomen along with the tumor thrombus.

Roux-en-Y anastomosis was performed. A side-to-side jejunojejunal anastomosis was created approximately 30 cm from the ligament of Treitz with the linear stapler. The entry hole for the jejunojejunal anastomosis was closed with a continuous 4-0 synthetic monofilament barbed suture. A side-to-side esophagojejunal anastomosis was created approximately 35 cm from the jejunojejunal anastomosis with the linear stapler. The entry hole of the esophagojejunal anastomosis was closed with the linear stapler (Fig. 3K, L). The total operative time and console time were 418 and 398 min, respectively. The estimated blood loss was less than 5 ml. The operation was performed by a surgeon (T.O.) who had experienced more than 100 robotic gastrectomies.

### Clinical outcomes

The patient resumed oral ingestion of water on postoperative day (POD) 2 and porridge on POD 3. Follow-up CT showed no PV obstruction. The drain tube was removed on POD 4. There were no complications. The patient was discharged on POD 9.

**Fig. 4:**
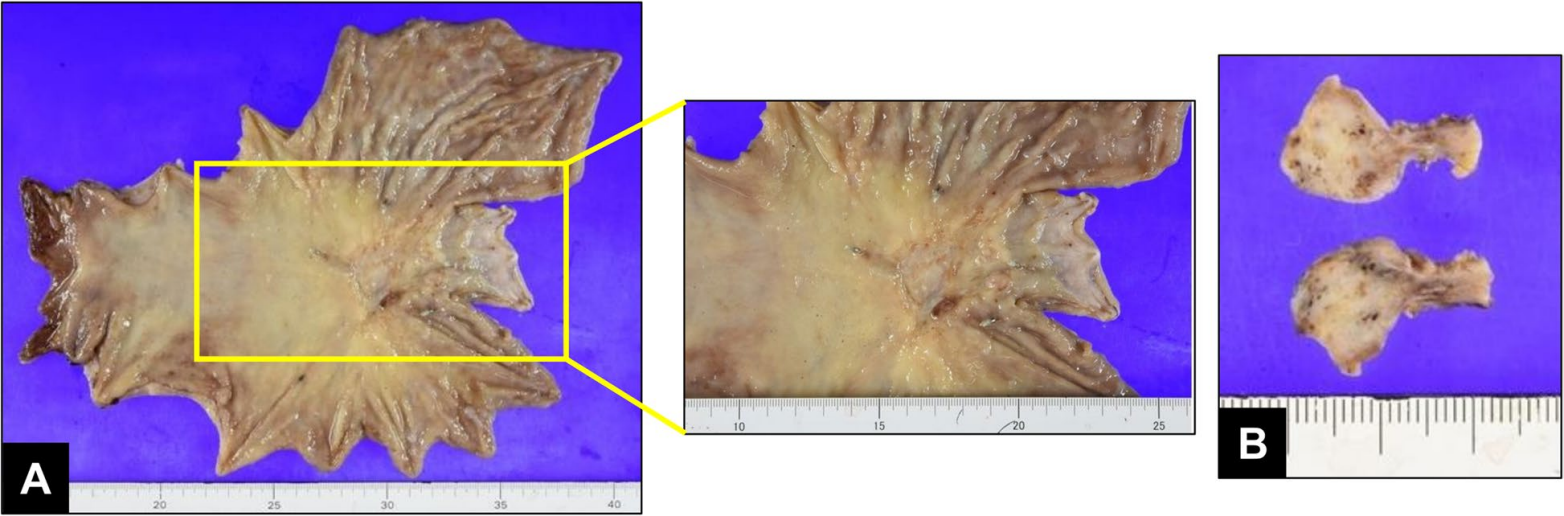
**A**, **B** Surgical specimen. Stomach (**A**) and portal vein tumor thrombus (**B**)

**Fig. 5:**
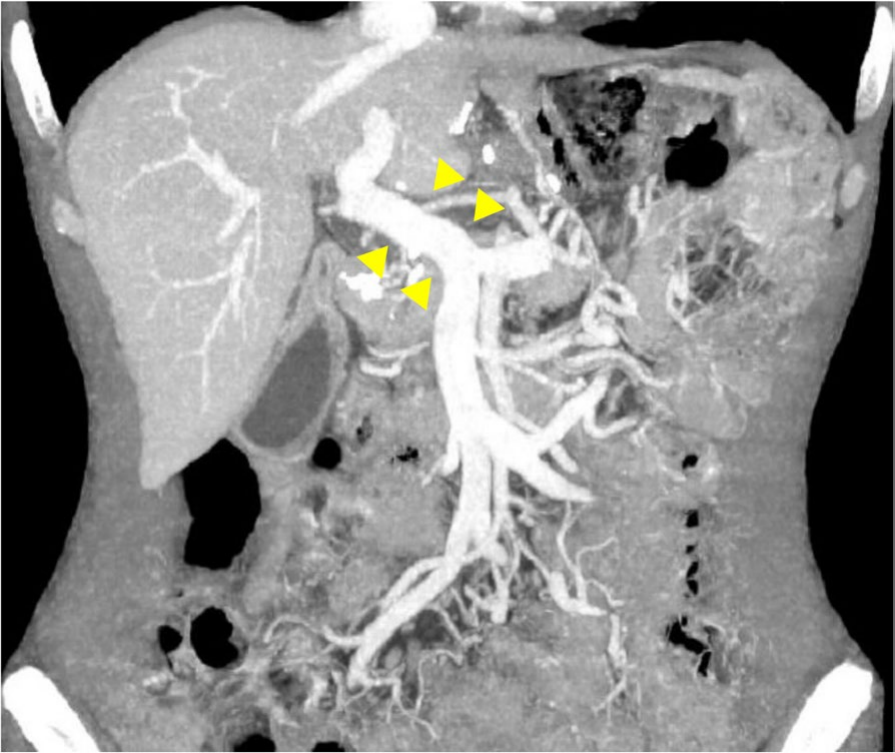
Computed tomography showed the disappearance of the portal vein tumor thrombus

Pathological examination revealed poorly differentiated adenocarcinoma (solid type) with invasion of the subserosa, perigastric lymph node metastases, and PVTT (Fig. 4A, B). The effectiveness of preoperative chemotherapy was pathologically diagnosed as Grade 2a, which means viable tumor cells remained in 1/10 to less than 1/3 of the neoplastic area, according to the tumor response criteria in the Japanese Classification of Gastric Carcinoma [10, 11]. PVTT appeared resolved on CT (Fig. 5). The patient had received trastuzumab, and no recurrence had been observed for 14 months after surgery. However, the lesion suspected of recurrence was detected at the dorsal side of anastomosis along the hemiazygous vein by CT and PET on the 14 months after surgery. Therefore, nanoparticle albumin-bound paclitaxel (nab-PTX) plus ramucirumab were given as the second regimen (nab-PTX, 80 mg/m^2^/day, intravenously, day 1, 8, 15; ramucirumab, 8 mg/m^2^/day, intravenously, day1, 15, followed by 7 days’ rest). He has been received nab-PTX plus ramucirumab and has remained alive for 30 months after surgery.

## Discussion and conclusions

This is the first report about a patient with gastric cancer and PVTT who underwent robotic gastrectomy with thrombectomy and PV reconstruction. The operation was successfully performed as planned. We hereby report some techniques for robotic surgery in patients with gastric cancer and PVTT.

Robotic surgery has many advantages for gastrectomy with thrombectomy compared with laparotomy or laparoscopy [12–16]. First, since the robot provides highly magnified 3-dimensional (3D) high-definition vision, which displays 3D stereoscopic images, surgeons have a better 3D view. Second, since the multiple articulations of the endo-wrist can allow the instruments to curve over an acutely angled and deep area, the robot is able to approach the area and contribute to precise lymphadenectomy and thrombectomy. Third, tremor reduction with robot can also achieve more precise and safer surgery than laparotomy or laparoscopy. Moreover, a minimally invasive and precise procedure with the robot may contribute to fewer complications and shorter hospital stays [16–19].

In general, gastric cancer with PVTT is considered advanced. The main treatment for gastric cancer with PVTT is chemotherapy. However, several recent reports have shown that surgery improves the prognosis of patients with gastric cancer and PVTT [2–4, 20, 21]. Moreover, neoadjuvant chemotherapy (NAC) was reported to play an important role in reducing the size of the tumor. Nakao et al. and Orii et al. reported that NAC for patients with gastric cancer and PVTT induces the disappearance of PVTT and makes radical surgery possible [3, 4]. In two patients, 3 to 4 cycles of a regimen containing S-1 plus cisplatin was given as NAC. In the present case, the patient received NAC consisting of capecitabine, oxaliplatin, and trastuzumab. The primary tumor and PVTT decreased in size, corresponding to PR.

Several reports have described patients who received chemotherapy consisting of S-1, S-1 plus paclitaxel, 5-fluorouracil (5-FU), or 5-FU plus cisplatin after NAC and radical surgery [3, 4, 20, 21]. In the present case, the patient received trastuzumab alone after surgery since he experienced adverse events during NAC. Although the long-term postoperative survival was reported to be 44–156 months after surgery, there have no reports about patients with PVTT who received trastuzumab after surgery [3, 4, 20, 21]. In the present case, the patient received postoperative chemotherapy and had no recurrences until 14 months after surgery. After recurrence, he received second-line chemotherapy and has remained alive 30 months after surgery. However, the most appropriate timing of surgical intervention or chemotherapy and chemotherapy duration before or after surgery have not been established yet. Although the main treatment for gastric cancer with PVTT is chemotherapy, the surgical intervention may be considered when it is considered that the chemotherapy is effective and radical resection is possible. Robotic surgery may contribute to be the radical resection because of minimally invasive and precise surgery. More investigation is warranted.

## Data Availability

The material supporting the conclusion of this review has been included within the article.

## Abbreviations

PVTT: Portal vein tumor thrombus
HER-2: Human epidermal growth factor receptor-2
CT: Computed tomography
PET: Positron emission tomography
PR: Partial response
RECIST: Response Evaluation Criteria in Solid Tumors
CTCAE: Common Terminology Criteria for Adverse Events
LGV: Left gastric vein
POD: Postoperative day
3D: 3-Dimensional
NAC: Neoadjuvant chemotherapy
5-FU: 5-Fluorouracil
